# Spending of HIV resources in Asia and Eastern Europe: systematic review reveals the need to shift funding allocations towards priority populations

**DOI:** 10.7448/IAS.17.1.18822

**Published:** 2014-02-25

**Authors:** Andrew P Craig, Hla-Hla Thein, Lei Zhang, Richard T Gray, Klara Henderson, David Wilson, Marelize Gorgens, David P Wilson

**Affiliations:** 1 The Kirby Institute, University of New South Wales, Sydney, NSW, Australia; 2 Dalla Lana School of Public Health, University of Toronto, Toronto, Canada; 3 World Bank Group Washington, DC, USA

**Keywords:** HIV, cost-benefit analyses, programme evaluation, systematic review, concentrated epidemics, Asia, Eastern Europe, cost-effectiveness

## Abstract

**Introduction:**

It is increasingly important to prioritize the most cost-effective HIV interventions. We sought to summarize the evidence on which types of interventions provide the best value for money in regions with concentrated HIV epidemics.

**Methods:**

We conducted a systematic review of peer-reviewed and grey literature reporting measurements of cost-effectiveness or cost-benefit for HIV/AIDS interventions in Asia and Eastern Europe. We also collated HIV/AIDS spending assessment data from case-study countries in the region.

**Results:**

We identified 91 studies for inclusion, 47 of which were from peer-reviewed journals. Generally, in concentrated settings, prevention of mother-to-child transmission programmes and prevention programmes targeting people who inject drugs and sex workers had lower incremental cost-effectiveness ratios than programmes aimed at the general population. The few studies evaluating programmes targeting men who have sex with men indicate moderate cost-effectiveness. Collation of prevention programme spending data from 12 countries in the region (none of which had generalized epidemics) indicated that resources for the general population/non-targeted was greater than 30% for eight countries and greater than 50% for five countries.

**Conclusions:**

There is a misalignment between national spending on HIV/AIDS responses and the most affected populations across the region. In concentrated epidemics, scarce funding should be directed more towards most-at-risk populations. Reaching consensus on general principles of cost-effectiveness of programmes by epidemic settings is difficult due to inconsistent evaluation approaches. Adopting a standard costing, impact evaluation, benefits calculation, analysis and reporting framework would enable cross comparisons and improve HIV resource prioritization and allocation.

## Introduction

Asia is the second most HIV-affected region of the world and Eastern Europe is the only region of the world in which HIV epidemics continue to increase [1]. These regions are not only geographically adjacent but share similar HIV epidemic features. HIV epidemics in Asia and Eastern Europe are concentrated among most-at-risk populations (MARPs), specifically among people who inject drugs (PWID) and sex workers (SW) and more recently in some countries also among men who have sex with men (MSM) [1].

Although responses to HIV epidemics in these regions have increased over the past decade, they have not controlled the spread of infection due to an inadequate coverage of populations most at risk. The increased response to HIV epidemics is largely due to substantial bilateral and multilateral donor investment in low- and middle-income countries across the region [2]. However, it is acknowledged that as this investment is withdrawing [3] it is becoming increasingly important to get more value for the available HIV money by prioritizing the most cost-effective HIV interventions. Allocating resources in the most effective way will reduce new infections and the morbidity and mortality caused by HIV.

HIV/AIDS intervention effectiveness evaluation and cost-effectiveness studies have become important analytical tools to understand what HIV investments have bought and which future allocation of funds is likeliest to result in the greatest epidemiological impact. The most comprehensive review of the cost-effectiveness of HIV programmes we identified was by Pattanaphesaj and Teerawattanon [4], who reviewed evidence specific for Thailand between 1997 and 2008. More specific reviews included Wolfe *et al*. [5], which reviewed the cost-effectiveness evidence of antiretroviral therapy (ART) for PWID, focusing on low- and middle-income countries. Galárraga *et al*. [6] reviewed relevant literature published in the years 2005–2008 for low- and middle-income countries and Sweeney *et al*. [7] considered studies that investigated the integration of HIV and AIDS services with other health services. Although these studies are not an exhaustive list of HIV cost-effectiveness reviews, the purview of recent broad-ranging reviews does end at 2008, reviews of subsequent periods concentrate on particular intervention types and we have not identified any reviews of all intervention types across a global region.

We conducted a systematic review of cost-effectiveness studies of HIV/AIDS programmes across Asia and Eastern Europe in order to identify evidence for which type of interventions offer the best value-for-money to address HIV epidemics in this region. To our knowledge, the current study is the broadest such review yet conducted. We also review National AIDS Spending Assessments from 12 case-study countries across the region to ascertain to what extent prevention spending is aligned with cost-effectiveness evidence.

## Methods

The criteria for a study to be included in the review were that the study considered an intervention to prevent HIV infection or reduce the burden of HIV, either in terms of health (as quantified by e.g. quality adjusted life-years (QALYs)) or in financial/economic terms; that the intervention occurred in Asia or Eastern Europe, or, if amalgamated results for a group of regions were presented, that a majority of the regions in the group were in Asia or Eastern Europe; and that the study reported at least one of the following: (1) cost per HIV infection averted, cost per disability adjusted life-year (DALY) averted, cost per QALY gained, cost per life-year saved or information that allowed simple calculation to produce one of these indicators; or (2) cost at which an intervention would be deemed cost-effective; or (3) cost savings; or (4) net present value, rate of return, or benefit-cost ratio. Our inclusion criteria meant that we included cost-effectiveness analyses (CEA), cost-benefit analyses (CBA) and cost-utility analyses (CUA), as well as other kinds of economic evaluation.

We searched the following databases: PubMed, EMBASE, LocatorPlus, EconLit, Tufts Medical Center CEA Registry. We also searched the World Bank Documents & Reports database, as well as those of the Asian Development Bank, UNAIDS, the Department for International Development UK, the International AIDS Vaccine Initiative, the International Partnership for Microbicides, the Office of Health Economics UK and PEPFAR. We also conducted Google searches on individuals known to have produced relevant papers or reports, models known to be used in HIV CEA and on each of the countries considered (the large numbers of results for these Google searches meant that checking each individually would have been prohibitively time-consuming: as such, we chose to check the first 100 results of each query). We adjusted the list of search keywords according to the capabilities of each search engine, but we required a match for a keyword synonymous with “HIV” or “AIDS,” and a keyword similar in meaning to “cost-effectiveness” or “programme evaluation.” We have provided the full list of search strings, the dates on which they were conducted and the number of hits (Supplementary file 1).

We also searched the references of identified studies and included referenced documents if they met our inclusion criteria. In addition, we included any relevant documents that we encountered for any reason during the course of the review. In several cases contacting an author with a request for further information also yielded documents that were considered for inclusion. Where evaluations in multiple studies considered the same intervention and were all conducted before the intervention or all conducted during/after the intervention, we chose one study for inclusion on the basis of comprehensiveness and publication date.

To better enable comparison of results from disparate countries and years we converted all incremental cost-effectiveness ratio (ICER) results into 2011 US$. Where ICERs were given in a non-US currency we converted the ratios into US$ for the same year, by dividing by the US per capita gross domestic product (GDP) purchasing power parity (PPP) and multiplying by the per capita GDP PPP corresponding to the non-US currency used. Per capita GDP PPP measures the value of goods produced in a country relative to the *in-country* purchasing power of that country's currency. By using PPP instead of the exchange rate to do the conversion from local currency to US$ we estimate the number of US$ that would buy similar goods in the United States as could be bought in the original country with the amount of local currency to be converted. Under this approach, when ICERs were given in a non-US currency that was also not the currency of the country in which the intervention took place, the conversion used the per capita GDP PPP corresponding to the country of the currency rather than the country of the intervention. Per capita GDP PPP were sourced from the International Monetary Fund [8] (Taiwan) and World Bank [9] (all others); the World Bank figures did not include per capita GDP PPP for 2012, so for those we used the corresponding 2011 values. We then inflated that value into 2011 US$ using medical care consumer price indexes taken from the United States Department of Labor [10]. In many cases, a study provided only US$ or international dollar ICERs; in these cases we skipped the currency conversion step.

When recording ICERs, we included ranges if these were noted alongside or in place of point estimates. We excluded ranges if they were noted in a separate sensitivity/uncertainty analysis section. Some studies that reported ICERs for a number of different interventions also calculated the ICERs of combinations of these interventions; in these cases, we reported only the ICERs for the separated interventions.

We standardized outcomes of studies for visualization and comparison purposes. Considering the World Health Organization (WHO)-CHOICE criteria for cost-effectiveness thresholds compare ICERs to a country's GDP [11], we divide the 2011 US$ ICERs by the 2011 per capita GDP (nominal) of the country in which the intervention was performed to normalize results. We used per capita GDP from [12] for Taiwan and [13] for all others. No per capita GDP values were available for the regions considered in studies that presented multi-country amalgamated results, and so these were not standardized for inclusion in figures.

We calculated summary statistics for the cost per HIV-infection-averted ICERs (in 2011 US$). To calculate these, we used the point estimates where available; where unavailable, we took the mean of the lower and upper bounds. For those studies in which ICER was recorded as “Cost-saving,” we treated the ICER as 0 (although the true ICER would have been negative).

We conducted a simple quality assessment of the included studies, using a slightly modified version of Neumann *et al*.'s checklist [14]. As part of this we calculated a “checklist success score” for each study: this was the percentage of non-N/A checklist items for which the result was not “no” or “unclear.”

In order to compare actual spending patterns to our findings on which populations can be targeted with HIV interventions in a cost-effective manner, we estimated for 12 countries the proportion of resources allocated to prevention programmes for SW/clients, MSM, PWID and the general population, using HIV spending and budgeting data [15–41] and communication with in-country stakeholders. Programmes without a clear priority population were designated as “Not targeted.” We excluded indirect costs including overhead or management costs and health infrastructure costs. Proportions allocated to each group were estimated from available spending data over the period 2007–2011. No adjustment for inflation was made.

## Results

**Figure 1:**
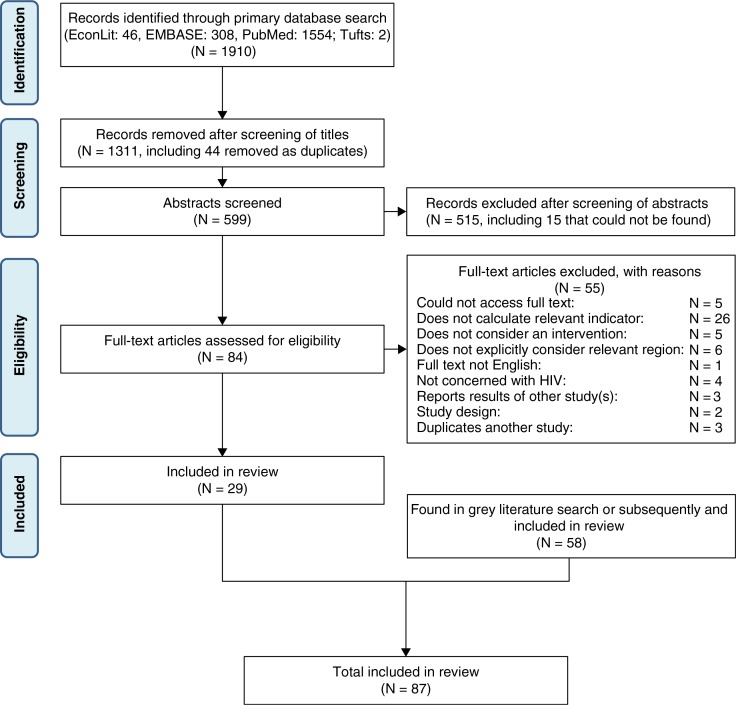
Flowchart indicating inclusion and exclusion of studies (with numbers of studies *N*) at each stage of the review process.

A flowchart of identified relevant studies and inclusions/exclusions according to different criteria is presented in Figure 1.

We included 91 studies (refs. 42–129 and J. Stover, personal communication, August 16, 2012; J. Bottcher, personal communication, August 17, 2012; D.P. Wilson, personal communication, November 28, 2012); these studies are summarized in a table (Supplementary file 3). Of the studies included, 47 were peer-reviewed journal publications. There were 28 countries considered individually; 9 studies gave amalgamated results for groups of countries. The country represented in the most studies was Thailand (21 studies), followed by India (16 studies) and Ukraine (7 studies). Of the 91 studies, 64 considered a single country/region and primary target group; the remainder compared multiple regions and/or primary target groups. The number of included studies, by region/country and primary target group of intervention(s) evaluated, is summarized in Table 1.

**Table 1:** Counts of studies reporting at least one intervention of given combination of region and primary target group

	Primary target group								
	
Region	PWID	HIV +	MSM	PMTCT	General population	SW/clients	MARP(s)	Mixed/TB + /unclear	Other vulnerable
East Asia									
China	5	1	1		2	1			
Hong Kong									
Japan					1				
Taiwan		1							
South Asia									
Bangladesh	1								
India		3	1	5	5	6		6	2
Nepal	1								
Pakistan	1							2	
Sri Lanka								1	
Southeast Asia									
Cambodia		2		1	2			3	
Indonesia	3	1		1	1			2	
Papua New Guinea		1		1	1			2	
Philippines									1
Singapore		1							
Thailand	2	8	3	6	10	2		5	1
Timor Leste		1			1			1	
Vietnam	1			1			1	1	
Southeast Asia Region B	1								
Southeast Asia Region D		1		1	1	1			
Central Asia									
Afghanistan								1	
Kazakhstan	1								
Tajikistan	1								
Uzbekistan	1								
Multiple, Central Asia	1		1			1		2	1
Eastern Europe									
Armenia	1								
Belarus	2								
Estonia	1								
Georgia	1								
Moldova	1							2	
Russia	1	1			1			1	
Ukraine	3	3	1	1				1	
Multiple, Eastern Europe	1								
Other									
Multiple, Asia	2		1		2	1		1	
Multiple, other	1	1	1	1	1	1		1	

We included programme evaluations of future/hypothetical interventions, as well as programme evaluations of in-progress/completed interventions: there were 65 of the former, 32 of the latter and 2 for which this was unclear. Eight studies included both before and after analyses. (Some studies considered in-progress or completed programmes, but analyzed cost-effectiveness for extensions or expansions of those programmes; we considered such evaluations to be future/hypothetical.) Of the evaluations of in-progress/completed studies, one assessed a randomized controlled trial [59], one compared patient outcomes before and after the introduction of highly active ART [81] and one compared different arms of an observational cohort [74]; the rest estimated effectiveness using approaches such as mathematical modelling.

Cost per HIV infection averted was the most-reported of the indicators we considered: 45 of the 91 studies gave at least one value for this indicator, for a total of 194 values with a mean of US$187,248, population standard deviation US$899,973, minimum cost-saving, first quartile US$567, median US$2,362, third quartile US$18,028 and maximum US$10,687,255.

Peer-reviewed journal publications performed better in the quality assessment (Supplementary file 2), with the mean checklist success score for peer-reviewed journal publications being 61% versus 32% for other studies.

Only one of the studies of an in-progress/completed intervention clearly stated that it was an evaluation planned from the outset, although many other evaluations were presumably in the same category even if they did not make that explicit. Of the studies of future/hypothetical interventions, 10 were clearly programme evaluations carried out during their planning phases—all were either World Bank or Asian Development Bank publications. A feasibility assessment by the WHO also could be added to that number.

We noted whether studies of in-progress/completed interventions used prevalence or incidence routine surveillance data in determining effectiveness; there were only two studies where the answer was an unequivocal yes. In the remaining cases, it was considered that the studies had not used such data, or that they were not clear on this point; however, many studies used mathematical models and it is possible that surveillance data were used for calibration without this being stated in the study.

Of the 32 studies that evaluated in-progress/completed interventions, in five cases the source of the cost data used was unclear. In each of the other 27 cases cost data were drawn from actual costs and/or other sources, although where these were not available costs were assumed.

**Figure 2:**
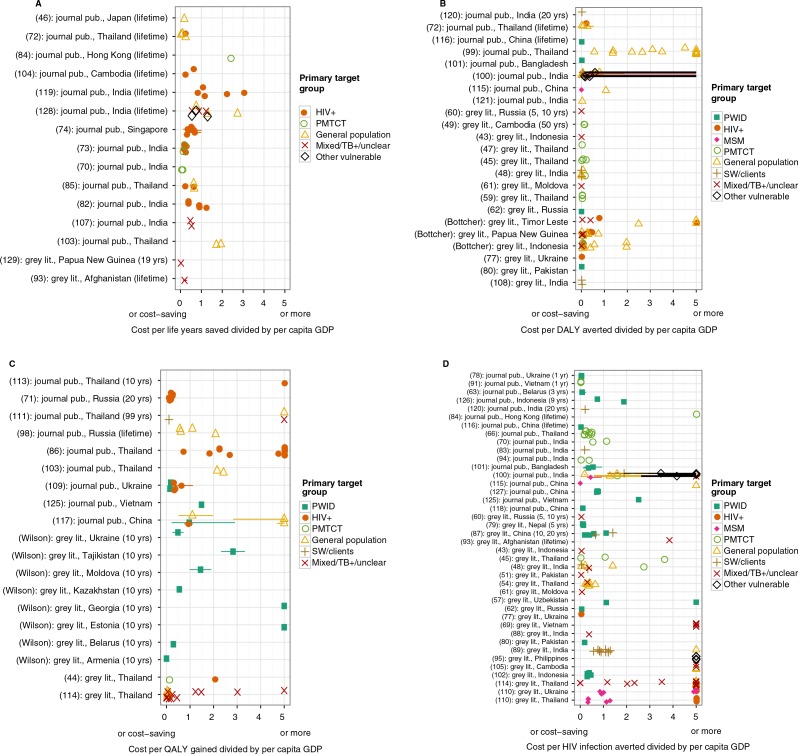
Point estimates and ranges for ICER divided by per capita GDP. Studies are included here if they reported ICER for an individual country (or if this could be easily calculated), and if the ICER comparator was “no intervention” or “status quo.” Numbers in brackets at the start of a label are the reference number; a name in brackets indicates that the study was received as a personal communication. Study timeframes, where known, are given in brackets at the end of the label. Many studies gave multiple values for a particular ICER, representing variations such as different coverage levels; all values are included in the figure. The ranges are those given in the studies; for the range meaning, see the summary table (Supplementary file 3). For clarity, if a study gave a range but no point estimate, the point estimate was considered to be the midpoint of the range. (A) ICER is cost per life-year saved. (B) ICER is cost per DALY saved. (C) ICER is cost per QALY gained. (D) ICER is cost per HIV infection averted.

The cost-effectiveness of HIV interventions varied substantially across the Asia/Eastern Europe region. A comparison of ratio estimates of ICERs/per capita GDP for all identified evaluated interventions is provided in Figure 2.

Many studies gave ICERs for a single programme incorporating a number of interventions. Therefore, results are differentiated by programme primary target group rather than by intervention type. Whether results are presented according to incremental cost per (a) life-year gained, (b) DALY saved, (c) QALY gained or (d) HIV infection averted, interventions appear to range from less than one per capita GDP to greater than 5 per capita GDP (Figure 2). Although there is variation in cost-effectiveness ratios for all targeted population group interventions, broadly it is identified that prevention of mother-to-child transmission (PMTCT) interventions and interventions targeted at PWID and SW/clients seem to have lower ICERs/per capita GDP, while programmes that were non-targeted or for the general population seem to have greater ICERs/per capita GDP (Figure 2). Relatively few studies focused on evaluating programmes targeting MSM. It is also important to note that there were large differences between studies in methodologies for assessing impacts and estimating costs (e.g. some studies considered only the unit costs of the intervention project, while others included infrastructure costs, while still others also included averted health care costs). This means that direct comparison of results across studies must be done with caution. Of particular importance is the large difference in time horizons considered in the included studies for assessment of benefits, which ranged from one year to lifetime. Some studies included the costs of health care while other studies did not. The most common form of annual discounting used was 3% for both costs and benefits, but this was not universal and there were studies in which costs and/or benefits were not discounted. Methods for estimating the burden avoided by the intervention evaluated varied from dynamic mathematical models to a simple assumption of the percentage of infections averted [51].

Of the 91 studies, 51 indicated whether or not the interventions studied were considered cost-effective and/or cost-saving, and 2 studies indicated costs at which the interventions would be considered cost-effective, based on HIV mobility [53] or vaccine costs [112]. No study reported that none of the interventions considered were cost-effective, although in many cases statements of cost-effectiveness were qualified with epidemic condition thresholds that would be necessary for the intervention to be cost-effective (e.g. [98]). The threshold or comparator for establishing cost-effectiveness varied: most used the WHO's standard, with interventions with a cost-effectiveness of less than the per capita GDP considered highly cost-effective, and those with a cost-effectiveness of between one and three times the per capita GDP considered cost-effective [11]. Other willingness-to-pay thresholds included medical costs for a person infected with HIV [62] and “a variety of formal and informal international standards” [84]. The particular ICER compared to the chosen willingness-to-pay threshold varied between studies and gross national income was sometimes used in place of GDP.

**Figure 3:**
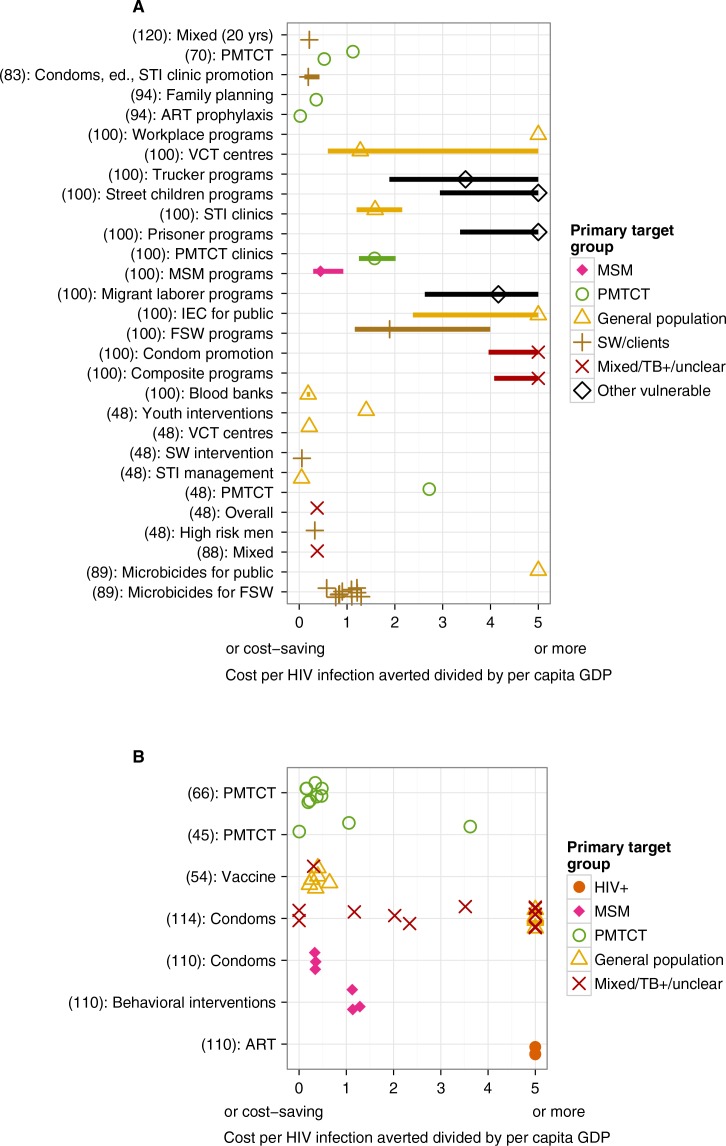
Point estimates and ranges for cost per HIV infection averted divided by per capita GDP, where this could be established and where the comparator was “no intervention” or “status quo,” for interventions in India (A) and Thailand (B). Study timeframes, where known, are given in brackets. Many studies gave multiple values for a particular ICER, representing variations such as different coverage levels; all values are included in the figure. The ranges are those given in the studies; for the range meaning, see the summary table (Supplementary file 3). For clarity, if a study gave a range but no point estimate, the point estimate was considered to be the midpoint of the range.

The two countries for which the greatest numbers of health economic evaluations have been conducted are India and Thailand. Findings from evaluations conducted in these countries further emphasize the message that targeted programmes are generally cost-effective whereas those aimed at the general population are not cost-effective. In Figure 3, the cost per infection averted divided by per capita GDP is shown for evaluations of programmes conducted in (A) India and (B) Thailand.

For India, non-targeted interventions or programmes for the general public, including workplace programs, information, education and communication (IEC), microbicide programmes for the public, mixed/tuberculosis (TB)+/unclear programs and programs for tuckers, street children, prisoners and migrant labourers had an ICER/per capita GDP ratio point estimate above 3. Some general population programmes were more cost-effective, including youth-based interventions, voluntary counselling and testing (VCT), sexually transmitted infection management (STI) and blood banks. Programmes targeting SW and programmes for PMTCT were deemed to be cost-effective in all evaluations. Similarly for Thailand, interventions targeting the public and mixed target groups have a wide range of ICER/per capita GDP ratios; notably, the most common interventions of condom distribution and education programmes for the public and mixed target groups have relatively poor cost-effectiveness. It was found that interventions targeting MSM have an ICER/per capita GDP ratio of less than 2, as does PMTCT in all but one evaluation. ART programmes were deemed to have a high ICER/per capita GDP ratio.

**Figure 4:**
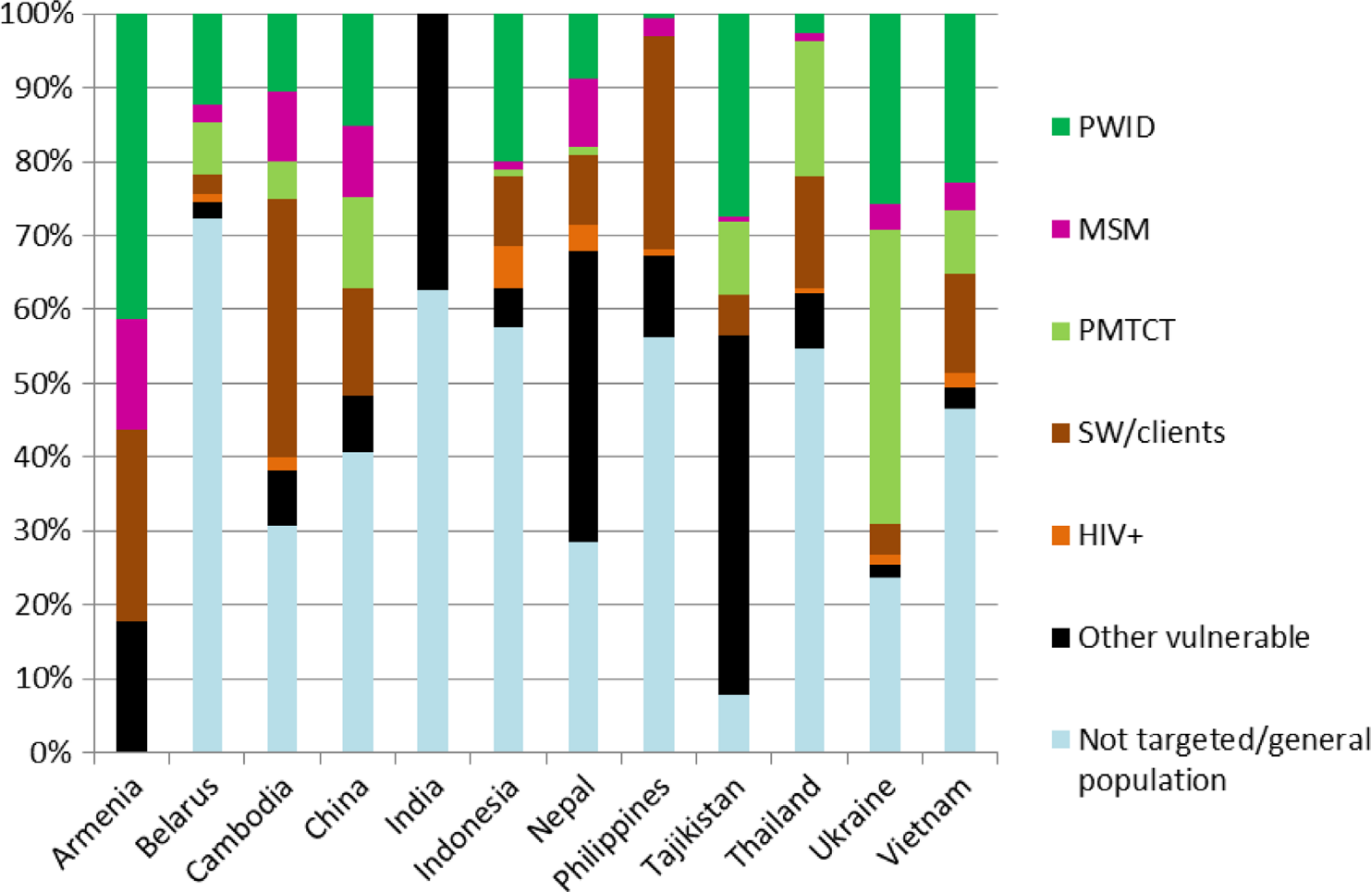
Allocation of 2007–2011 prevention programme spending by country and broad target population group. The “Other vulnerable” category includes programmes targeting unspecified MARPs.

A relatively large proportion of HIV prevention resources are allocated to the general public or otherwise untargeted. The allocation of prevention programme spending to different target groups is given for 12 countries in Figure 4.

There is large variability in the proportions of resources allocated to different target population groups. However, greater than 30% of all HIV resources were provided for the general population or non-targeted in eight of twelve countries and greater than 50% of HIV resources were non-targeted in five countries (Figure 4).

## Discussion

To determine whether actual spending on HIV interventions is in accordance with the latest evidence of HIV intervention cost-effectiveness, and also to address the lack of a recent comprehensive review of such evidence from countries with concentrated epidemics, we conducted a systematic review of studies of cost-effectiveness of HIV interventions in Asia and Eastern Europe.

Generally, we found that programmes targeting populations at highest risk, such as PWID, SW and MSM, were most cost-effective while programmes targeting the general public were not cost-effective or much less cost-effective than targeted programmes. However, for all target groups some evaluated programmes reported low ICERs and others reported high ICERs. Some programmes for the general population, such as VCT and STI treatment programmes, were shown to have greater cost-effectiveness (in India) than other programmes for the general public. Although VCT and STI programmes do target the public, their users will self-select and be those who consider themselves to be at risk of acquiring HIV or other STIs. Therefore, these more cost-effective “general population” programmes are also targeted towards those at greater risk. Conversely, workplace programmes, IEC and other non-targeted programmes are somewhat indiscriminate in that they will cover many people who are at low risk for HIV infection. This is likely to be the main reason for these broader programmes’ lower cost-effectiveness.

We also determined, through collation of data from National AIDS Spending Assessments from twelve countries in the region, that for eight of these countries, over 30% (and as high as 72%) of prevention funding over recent years was non-targeted and/or allocated to the general population despite the evidence of the low cost-effectiveness of these programmes and that more cost-effective programmes for most at-risk populations are generally far from saturation [41, 130].

It could be considered that a priority is to guard the general population from the entry of HIV that would otherwise spark generalized epidemics as seen in Southern Africa. However, there is little evidence of a generalized epidemic occurring to date. The most at-risk populations of PWID, SW and MSM are often marginalized and therefore it may be politically difficult to invest significantly in health interventions targeting them. However, the evidence collated here suggests that decision makers would be wise to shift the limited HIV/AIDS resources available away from general population programmes and towards interventions that specifically target groups of people at greatest risk of infection. The interventions implemented should be those that have proven efficacy and are feasible in the given contexts. In doing so, the investment has the potential to make the greatest epidemiological impact and future economic return on the given investment.

A recent study found that a trial of antiretroviral pre-exposure prophylaxis (PrEP) given to PWID in Bangkok, Thailand, reduced transmission of HIV by 48.9% [131]. We have seen in this review that interventions targeting PWID can be very cost-effective and so look forward to a cost-effectiveness evaluation of PrEP for PWID. PrEP has also reduced transmission by 44% in MSM [132], and so it may be that PrEP becomes a key HIV intervention for many at-risk sub-populations. A recent review of CEA of treatment strategies for persons living with HIV/AIDS (PLWHA) found that increasing access to ART was generally more cost-effective than investing in more laboratory monitoring for those on ART [133]. Most of the studies included in that review considered Africa, but in those studies in our review that assessed interventions that primarily target PLWHA, ART seems to have been generally more cost-effective than alternative PLWHA-targeted interventions (such as TB interventions); thus, our findings broadly agree.

Many studies (51 of 91) made their own assessment as to whether an intervention was cost-effective or cost-saving. We attempted to standardize comparison between studies by reporting ICERs and ICERs/per capita GDP. The range of outcome measures used (HIV infections averted, DALYs averted, QALYs gained, life-years saved) further complicates comparison. The most-reported of these indicators, cost per HIV infection averted, is only reported by 43 of the 91 studies—less than half. There were also little-to-no similarities in the way in which the different studies were carried out. There is a lack of a standard in approaches to measuring effectiveness, costing and assessing cost-effectiveness, as well as in the time horizon over which analyses are conducted. The lack of standardization has been highlighted in the literature (e.g. [6] and [134]). The difference between assessing effectiveness over one year and over a lifetime can be great, as is the difference between including and excluding medical costs that would have been incurred as the result of contracting HIV, yet there were many such variations in the measurement approaches used in the studies we reviewed. These variations reduce the utility of comparing ICERs reported by different studies and should be seriously considered when interpreting the findings of this review. Indeed, such variations make it difficult for any attempt by decision makers to evaluate and implement best evidence-based practice. That all studies declared cost-effectiveness or found at least one of the intervention components cost-effective may also indicate bias in scientific approach or publication bias. To reduce this potential, we recommend that a registry of CEA protocols be established, similar to ClinicalTrials.gov [135] for clinical trials, and results presented as per the protocol regardless of the finding.

We recommend the adoption of a consistent costing and reporting framework, to better enable the comparison of the findings of different studies and to reduce the potential for methods of measuring costs and benefits to be selectively chosen in the interests of calculating a favourable cost-effectiveness. Guidelines on clear calculation of cost-effectiveness have existed for some time (e.g. [136–139]) but [140] noted that guidelines do not necessarily agree with one another, and that their recommendations do not always provide sufficient detail as to how they should be followed. In addition, the emphasis generally seems to be on promoting clarity (i.e. by being explicit about what has been included and excluded in cost and benefit calculations) rather than on proscribing what should be included and excluded. Proscriptive guidelines would be more effective in creating a consistent and comparable body of evaluation literature. As a starting point for a proscriptive set of guidelines, we recommend: healthcare costs saved be included when costing; the “lifetime” timeframe (which was the most frequently used in the studies in this review) be used; both economic and financial costs be reported; costs and cost-effectiveness ratios be reported in local currency, US$ (converted using standard exchange rates) and international dollars; HIV infections averted (where this makes sense) and QALYs gained be used as the measures of benefit; and point estimates as well as 95% credible intervals be given for ICERs. For all of these suggestions, there are arguments for using an alternative measure or method, and discussion should be made before guidelines are decided upon, but what we consider important is not so much *which* particular method is recommended in future guidelines, but simply that *a* particular method is recommended.

Almost half of the studies included in this review were not from peer-reviewed journal publications, and the results of our quality assessment suggest that the standard of reporting in peer-reviewed journal publications is higher. It should be noted that there were many ambiguous cases for even seemingly open-and-shut checklist items and so there was a large degree of subjectivity involved in the assessment. Also, the results are to a large extent a measure of how much information was directly available to the reader, and so the low rating of short documents, including conference abstracts and posters, is unavoidable. Given the difference in rated reporting quality of the peer-reviewed and grey literature, it is important to note whether there is a noticeable difference in their broad findings. Figure 2 identifies whether or not each study shown was peer reviewed; we do not consider there to be a clear pattern to the cost-effectiveness ratios based on whether or not the source was peer reviewed, and so we believe it is reasonable to draw conclusions by considering together the peer-reviewed and grey literature.

This study has some limitations. The National AIDS Spending Assessments represented the best indication we could find of actual spending on HIV interventions, but it should be noted that spending categorized in Figure 4 as “not targeted” may have been targeted at high-risk groups. Also, our exclusion of separate sensitivity/uncertainty analysis sections reported within reviewed studies means that some information that is in the literature was not included in our review. Although we attempted to restrict study inclusion to one per intervention, the high proportion of evaluations of future/hypothetical interventions means that results of some studies for the same region/country will probably have some overlap with other evaluation studies. We did not include a restriction on publication date in our inclusion criteria and, therefore, some of the included studies are relatively old. Results from these studies should be viewed with more caution because the cost of some interventions may have changed (e.g. ART). Furthermore, the epidemic dynamics from different time periods, and in different country settings, may influence the cost-effectiveness of interventions. Studies that compare different intervention types and/or interventions in different regions are valuable because they provide comparison of those interventions without the usual concern about different methods and settings, allowing for better dissemination of knowledge and for general conclusions and principles to be elicited, which can inform decision-making.

Most of the studies included (65 of 91) considered only one relevant region/intervention combination. More studies contrasting multiple regions and/or interventions would be valuable. Of particular benefit might be more investigations that contrast the cost-effectiveness of different interventions targeting PWID, SW/clients, individuals living with HIV, and the public. As can be seen in Table 1, several regions are considered by only one study, while only interventions in Thailand and India have each been considered in a relatively large number of studies. There are also regions within Asia and Eastern Europe not represented or under-represented in the literature. There is also a lack of investigation into the effectiveness and cost-effectiveness of programmes primarily targeting MSM.

With around one third of included studies evaluating in-progress/completed evaluations, our results are dominated by future/hypothetical studies that project estimated cost-effectiveness of future programme implementation. Assessing the potential cost-effectiveness of different budget decisions and also evaluating interventions after implementation may provide greater rigor to the process of identifying greatest value for money. However, in current environments where decisions need to be made on resource prioritization, our study suggests the greatest value for money, resulting in largest epidemiological impact, will be attained by targeting populations and sub-populations of people at greatest per capita risk of infection. We suggest that less-targeted intervention programmes should be considered only when these groups are covered with programmes towards saturation.
